# Sunshine Act expansion and the prescription of generic medications by advanced practice providers

**DOI:** 10.1093/haschl/qxag004

**Published:** 2026-01-10

**Authors:** Hasan Nadeem, Jing Li, Lucas M Donovan, Laura C Feemster, David H Au, Kevin I Duan

**Affiliations:** Division of Pulmonary, Critical Care, and Sleep Medicine, University of Washington School of Medicine, Seattle, WA 98195-6522, United States; The Comparative Health Outcomes, Policy, and Economics (CHOICE) Institute, Department of Pharmacy, University of Washington, Seattle, WA 98195-7630, United States; Division of Pulmonary, Critical Care, and Sleep Medicine, University of Washington School of Medicine, Seattle, WA 98195-6522, United States; Center of Innovation for Veteran-Centered and Value-Driven Care, Veterans Affairs Puget Sound Health Care System, Seattle, WA 98108, United States; Division of Pulmonary, Critical Care, and Sleep Medicine, University of Washington School of Medicine, Seattle, WA 98195-6522, United States; Center of Innovation for Veteran-Centered and Value-Driven Care, Veterans Affairs Puget Sound Health Care System, Seattle, WA 98108, United States; Division of Pulmonary, Critical Care, and Sleep Medicine, University of Washington School of Medicine, Seattle, WA 98195-6522, United States; Center of Innovation for Veteran-Centered and Value-Driven Care, Veterans Affairs Puget Sound Health Care System, Seattle, WA 98108, United States; Division of Pulmonary, Critical Care, and Sleep Medicine, University of Washington School of Medicine, Seattle, WA 98195-6522, United States; Division of Respiratory Medicine, University of British Columbia, Vancouver, BC V5Z 1M9, Canada; Centre for Lung Health, Vancouver Coastal Health Research Institute, Vancouver, BC V5Z 1M9, Canada

**Keywords:** health policy, Sunshine Act, transparency, generic medications

## Introduction

The Physician Payments Sunshine Act requires the public disclosure of payments made to clinicians from drug and device manufacturers in the United States, with the goal of increasing transparency that could potentially improve drug costs by encouraging generic prescribing over brand-name prescribing. Prior studies of the transparency law's implementation in 2013 among physicians have demonstrated an association with lower brand-name prescriptions, but were limited to certain drug classes and geographic areas.^1^ In 2021, the Sunshine Act expanded to include reporting of industry payments to advanced practice providers (APPs), a rapidly growing group of prescribers.^2^ We leveraged the policy expansion to conduct a nationwide, quasi-experimental study to assess whether the policy's implementation changed prescribing behavior among physician assistants, nurse practitioners, and clinical nurse specialists—clinicians previously excluded from reporting.

## Methods

We utilized publicly available, provider-level Medicare Part D prescribing data^3^ linked to Open Payments data,^4^ from January 1, 2016, to December 31, 2022, to conduct a difference-in-differences (DID) analysis. National Provider Identifier numbers were used to link clinician prescription data to Open Payments data on “general” and “research” payments. We defined APPs as the treatment group subject to Sunshine Act expansion on January 1, 2021. We defined physicians as the control group since they were unaffected by the 2021 expansion. The Open Payments Program, which catalogs industry payments to clinicians, defines APPs as follows: physician assistants, nurse practitioners, clinical nurse specialists, certified registered nurse anesthetists and anesthesiology assistants (CRNAs), and certified nurse-midwives (CNMs). We focused on long-term, outpatient prescribers, and therefore excluded CRNAs and CNMs since these providers typically provide episodic care with limited outpatient prescriptions.

The primary outcome was the clinician-specific, annual proportion of total prescription claims that were generic. Generic claims are defined in Medicare data using Food and Drug Administration (FDA) categorizations. To conduct our DID analysis, we used a fixed-effects linear regression model with clinician and year fixed effects and group-specific linear time trends. We adjusted for patient case mix using the average Hierarchical Conditions Category risk score of beneficiaries at the prescriber level. Standard errors were clustered at the clinician-level, accounting for serial correlation over time within the same clinician. We also conducted 3 sensitivity analyses: (1) an alternative control group of physicians who received no industry payments, (2) an expanded study period to include the initial years in which Open Payments collected data, and (3) inclusion of all APP types. We evaluated parallel trends visually (Figure S1), using linear regression (Table S1) and an event study plot (Figure S2). While visual inspection shows relatively parallel trends (Figure S1), linear regression and the event study plot demonstrated violation of parallel trends that was small in magnitude, but statistically significant, likely related to our large sample size. Therefore, we included group-specific linear time trends to control for baseline differential trends in generic prescription over the study period between APPs and physicians (Figure S3). Additional methodologic details are available in the Supplemental Methods. Analyses were conducted using Stata version 19.5 (StataCorp, College Station, TX). This study did not require institutional review board review as it used publicly available data.

## Results

Our sample included 1 252 427 unique clinicians, of whom 440 136 (35.14%) were APPs (Figure S4). Among active Medicare Part D prescribers, 52.7% of APPs received at least 1 industry payment, compared with 73.7% of physicians. Among those receiving payments, APPs received an average of $501.56 per year, compared with $2697.42 per year for physicians. There were 10.1 billion Medicare Part D claims from all providers across our study period, of which 1.9 billion (18.8%) were prescribed by APPs. The proportion of generic claims in 2016 was 83.7% for APPs and 80.9% for physicians and increased to 87.4% and 85.6% in 2022, respectively. Figure S1 plots trends in generic claims from 2016 through 2022. In our primary analysis, we found that expansion of the Sunshine Act in 2021 was associated with a 0.23 percentage point (ppt) (95% CI, 0.19–0.27 ppt) higher mean proportion of generic prescriptions by APPs, relative to physicians. Sensitivity analyses yielded similar effects (Table 1).

**Table 1.** DID results of the association of the Sunshine Act advanced practice provider expansion with generic prescriptions.

Model	Mean generic prescriptions, unadjusted raw estimates, %				DID estimate,^a^ percentage points (95% CI)
	Physicians (control)		Advanced practice providers (treated)		
	Prepolicy	Postpolicy	Prepolicy	Postpolicy	
Primary analysis	82.68	85.26	85.00	87.27	0.23 (0.19, 0.27)
Sensitivity analysis, MD, no payment	82.68	87.24	85.00	87.27	0.38 (0.32, 0.45)
Sensitivity analysis, all APP types	82.68	85.26	84.98	87.27	0.23 (0.19, 0.27)
Sensitivity analysis, 2013–2022 study period	81.29	85.26	83.94	87.27	0.17 (0.13, 0.21)

^a^DID estimate represents the percentage point change in the average proportion of generic prescriptions among advanced practice providers, relative to physicians after Sunshine Act policy expansion.

Abbreviations: APP, advanced practice provider; DID, difference-in-differences.

## Discussion

We found that expansion of the Sunshine Act to report industry payments to APPs was associated with a marginally higher, and likely clinically insignificant, proportion of generic prescriptions by APPs after policy implementation compared with physicians. There are several possible explanations for our findings. First, the policy expansion for APPs occurred within the context of a secular trend of increasing generic prescription for both physicians and APPs, which may have mitigated the policy effect on APPs specifically. The secular trend of greater generic prescribing in the United States could be explained by numerous simultaneous policy factors, including generic substitution laws, changes to the FDA generic approval process, and the impact of the original Sunshine Act implementation for physicians in 2013.^5^ It is important to note that our DID design addresses these policy factors by accounting for secular trends in prescribing. Second, while our overall results showed no major policy effect, it is possible that the Sunshine Act expansion had an effect in specific subgroups. For example, high-volume prescribers, high-industry payment recipients, or certain drug classes, such as those with available generic alternatives, could be differentially affected. This was beyond the scope of this study, which focused on the overall average effect, but warrants further investigation. Third, industry payments to APPs differ in type and value than those to physicians, which could also affect the susceptibility of APPs to the desired policy effect.^6^

Limitations include generalizability outside of Medicare, spillover effects from the varied spectrum of APP practice environments (ie, supervised, shared-practice, independent, etc), and possible residual confounding despite the quasi-experimental design. Furthermore, because the policy expanded during the COVID-19 pandemic, it is unclear whether supply-chain disruptions or the accelerated adoption of telehealth differentially impacted APPs and physicians, potentially influencing our results.

In conclusion, our study found that the Sunshine Act expansion did not have a clinically significant effect on generic prescribing by APPs in the United States. Further research is needed to evaluate whether transparency reporting may affect specific clinician subgroups or drug classes differently. Our findings have implications for jurisdictions outside the United States that may be considering transparency laws, suggesting that the marginal benefit of transparency may be limited when multiple policies already encourage generic prescribing and generic prescribing rates are high at baseline. Additional policy tools beyond transparency laws are needed to further lower drug costs in the United States.

## Supplementary Material

qxag004_Supplementary_Data

## Data Availability

Data are publicly available for download from the US Centers for Medicare and Medicaid Services website.
