# Supplementary material for: Sunshine Act expansion and the prescription of generic medications by advanced practice providers
Source: Health Aff Sch. 2026 Jan 10;4(1):qxag004. doi: 10.1093/haschl/qxag004 (PMC12822597; doi:10.1093/haschl/qxag004)
Supplement: qxag004_Supplementary_Data [file qxag004_supplementary_data.zip › Supplement SunshineAct_HealthAffairsScholar V4.docx]

**Online Supplement: Sunshine Act Expansion and the Prescription of Generic Medications by Advanced Practice Providers**

*Hasan Nadeem, MD, Jing Li, PhD, Lucas M. Donovan, MD, MS, Laura C. Feemster, MD, MS, David H. Au, MD, MS, Kevin I. Duan, MD, MS*

**SUPPLEMENTAL METHODS**

**Provider Classification:**

Advanced Practice Practitioners (APPs) and physicians were identified using the “Provider Specialty Type” and “Provider Credentials” variables. These variables are free text entries in the Medicare Part D dataset, abstracted from the National Plan and Provider Enumeration System (NPPES). To classify patients, we used the following approach. First, text entries from the “Provider Type” variable that clearly identified provider categories, such as “Nurse Practitioner,” “Physician Assistant,” or “Midwife,” were assigned to the APP group. Similarly, “Physician” or “Doctor” were assigned to the physician group. Next, we manually reviewed all entry types in the “Prescriber Credentials” variable to assign unique entries to the correct corresponding group. Entries such as “MD,” “DO,” and “MBBS,” were designated to the physician group. Similarly, entries such as “NP,” “DNP,” “PA,” “PA-C,” etc. were assigned to the APP group. Providers in the dataset that could not be accurately categorized based on available information were excluded. Please see the study flow diagram for additional details (**Figure S4**). Of note, the Open Payments Program and Medicare data refer to physician assistants, nurse practitioners, certified nurse specialists, and certified registered nurse anesthetists and anesthesiology assistants, and certified nurse midwives as “Non-Physician Practitioners.” In our study we utilize the term of “Advanced Practice Practitioners” in place of “Non-Physician Practitioners.”

**Measurement of the Primary Outcome:**

The outcome variable in our study is the annual proportion of total prescription claims that were generic. This specific outcome was selected to minimize missingness within the branded drug category. Missing claims for generic drugs and brand-name drugs occurred due to suppression of claims data to preserve the privacy of beneficiaries of very low volume prescribers (between 1 and 10 prescription claims of either generic or branded drugs). The degree of claims suppression was substantially lower for generic drugs (~230,000 observations) compared to branded drugs (~4 million suppressed observations). Therefore, calculating the proportion of total claims that were generic (as opposed to branded) led to fewer missing outcomes. There was no missingness or suppression of the total claims variable.

**Difference-in-Differences Methodology:**

We evaluated parallel trends both visually and using linear regression. While visual inspection shows relatively parallel trends (**Figure S1**), linear regression and an event study plots demonstrated violation of parallel trends (see **Table S1 and Figure S2**) that is small in magnitude, but statistically significant, likely related to our large sample size. Therefore, we included group-specific linear time trends to control for baseline differential trends in generic prescription over the study period between APPs and physicians. Inclusion of group-specific time trends in the pre-policy period is an established method to address non-parallel trends.^1^ After including group-specific linear time trends, a repeat event study plot demonstrated that parallel trends in the pre-policy period (**Figure S3**).

**Figure S1. Proportion of Generic Claims to Total Claims for Advanced Practice Providers and Physicians from 2016-2022
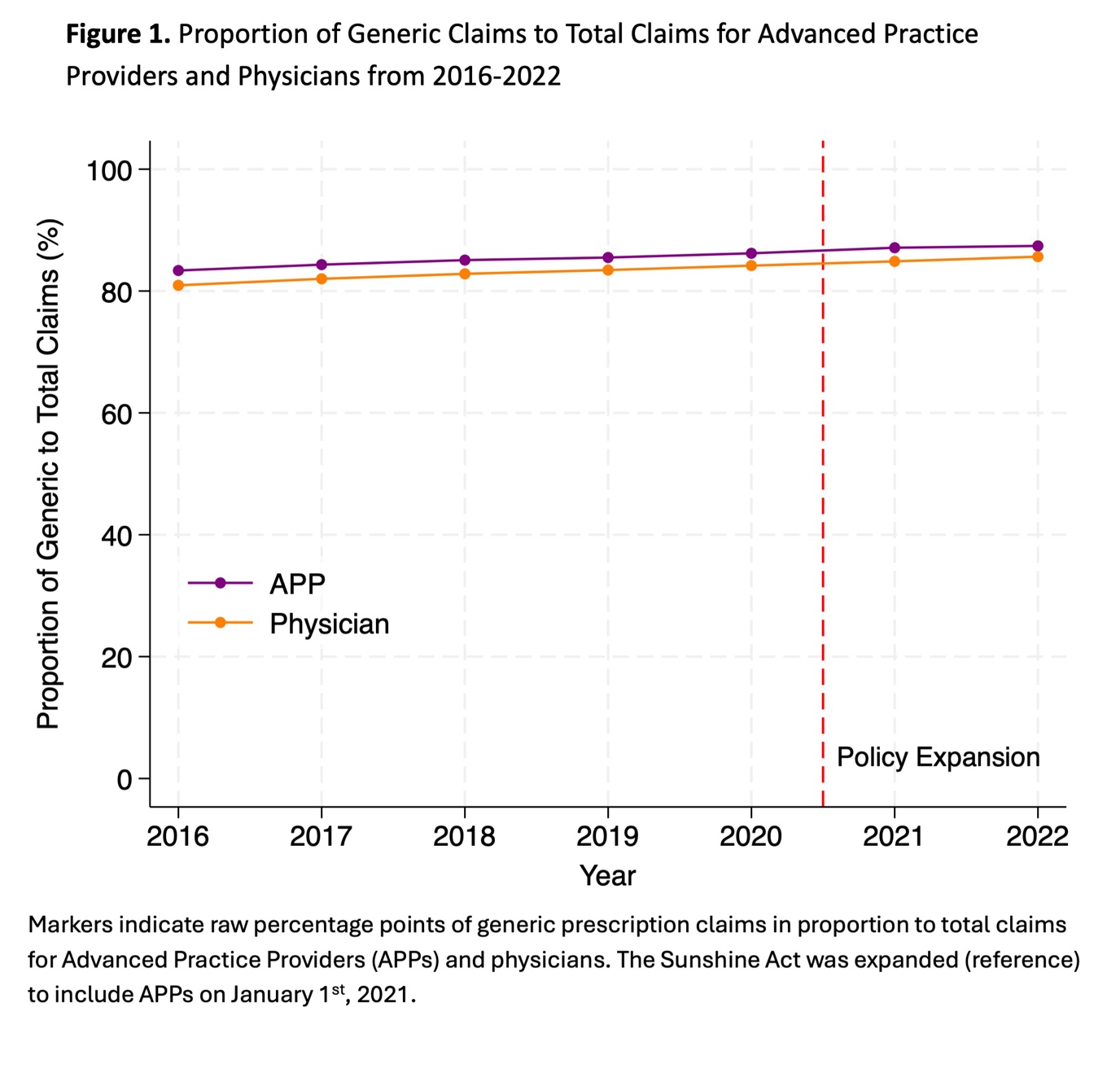
**

**Table S1:** Tests for Parallel Trends Assumption

| Variable | | | Coefficient (%) | 95% CI Low (%) | p-value |
| --- | --- | --- | --- | --- | --- |
|  | | |  |  |  |
| Interaction: *Provider Type**Year | | |  |  |  |
| APP*2016 | | | 0.76 | 0.71 - 0.82 | <0.001 |
| APP*2017 | | | 0.56 | 0.51 - 0.60 | <0.001 |
| APP*2018 | | | 0.42 | 0.38 - 0.46 | <0.001 |
| APP*2019 | | | 0.14 | 0.11 - 0.17 | <0.001 |
| APP*2020 | | | Ref | -  Abbreviations: Advanced Practice Practitioner, APP. Year prior to policy expansion (2020) selected as reference year. Coefficients and confidence intervals multiplied by 100 to reflect percentage points. | - |
|  |  |  |  |  |  |

**Figure S2: Event study plot to visualize for pre-policy parallel trends**

**Figure S3: Event study plot to visualize for pre-policy parallel trends after adjusting for group-specific linear time trends**

**Figure S4: Provider Study Flow Diagram**

**APP Sample**
(n = 440,136)

**Physicians Sample**
(n = 812,291)

Total APPs
(n = 446,790)

Medicare Part D Total Providers from 2016-2022
 (n = 1,708,853)

APP and Physician Provider Sample 2016-2022
 (n = 1,259,081)

Excluded (n = 449,772)

Neither APP or Physician Provider Credentials
(n = 387,692)

Missing Provider Credentials
(n = 43,962)

Changed Provider Credentials
(n = 185)

Suppressed Generic Claims
(n = 17,933)

Excluded (n = 6,654)

CRNA (n = 990)

CNM (n = 5,664)

**References:**

1. Angrist JD, Pischke JS. *Mastering ’Metrics: The Path from Cause to Effect*. Princeton University Press; 2014. Accessed May 10, 2025. https://press.princeton.edu/books/paperback/9780691152844/mastering-metrics
